# Regulation of Dietary Lipid Sources on Tissue Lipid Classes and Mitochondrial Energy Metabolism of Juvenile Swimming Crab, *Portunus trituberculatus*

**DOI:** 10.3389/fphys.2019.00454

**Published:** 2019-04-24

**Authors:** Ye Yuan, Peng Sun, Min Jin, Xuexi Wang, Qicun Zhou

**Affiliations:** Laboratory of Fish and Shellfish Nutrition, School of Marine Sciences, Ningbo University, Ningbo, China

**Keywords:** *Portunus trituberculatus*, krill oil, fatty acid, anabolism, β-oxidation, mitochondria, sirtuin, electron transport chain

## Abstract

An 8-weeks feeding trial with swimming crab, *Portunus trituberculatus*, was conducted to investigate the effects of different dietary lipid sources on the lipid classes, lipid metabolism, and mitochondrial energy metabolism relevant genes expression. Six isonitrogenous and isolipidic experimental diets were formulated to contain fish oil (FO), krill oil (KO), palm oil (PO), rapeseed oil (RO), soybean oil (SO), and linseed oil (LO), respectively. A total of 270 swimming crab juveniles (initial weight 5.43 ± 0.03 g) were randomly divided into six diets with three replications, each consisted of 45 juvenile crabs. The results revealed that crabs fed KO had highest lipid content in hepatopancreas and free fatty acids in serum among all diets. The anabolic pathway relevant genes: *fas* and *acc* were up-regulated in KO diet. The catabolic pathway relevant genes, *hsl*, was up-regulated in LO diet, while *cpt1* was up-regulated in KO diet. Whereas, the genes involved in the transport and uptake of fatty acids such as *fabp1* and *fatp4* were down-regulated in crab fed PO and RO diets. Furthermore, the gene expression levels of transcription factors: *srebp-1* and *hnf4*α in KO and SO diets were the highest among all diets. FO and KO diets had significantly higher unsaturation index of mitochondrial membrane than others. The genes related to mitochondrial energy metabolism, such as *Atpase6*, *sirt1*, and *sirt3* were significantly up-regulated in KO and SO diets. In summary, dietary KO and SO supplementation could improve the lipid metabolism, promote energy production for juvenile swimming crab and improve physiological process and function including molting. These findings could contribute to deepen the understanding of the physiological metabolism of dietary fatty acids for swimming crab.

## Introduction

Hepatopancreas, a key organ for absorption and storage of nutrients in various crustaceans, plays an important role in lipid metabolism, nutritional status and energy storage (Wang et al., 2008, 2014). The hepatopancreas stores a large amount of energy-supplying substances, especially lipids, to prepare for energy production and expenditure during molting, starvation, reproduction, limb regeneration, and other life activities (Huang et al., 2015). Therefore, the hepatopancreas is an ideal organ to study lipid metabolism and energy metabolism during the growth stage in crustaceans.

Lipid plays two major roles, as an important energy supply and as a source of essential fatty acids (EFA) for membrane integrity, which is the main organic reserve regulating the physiological metabolism of many crustacean species, especially the synthetic process of crustacean molting hormones (O’Connor and Gilbert, 1968; Harrison, 1990).

Over the last few decades, due to the effective supply of energy and sufficient levels of EFA, especially adequate omega-3 polyunsaturated fatty acids (n-3 PUFA), fish oil (FO) has become the foremost lipid source used in the feed for most species in aquaculture industry including crustaceans traditionally (Tocher, 2015). However, the global fish oil production may be insufficient to meet future demand in aquaculture owing to sustained reliance on marine fish oil and its own finitude (Betancor et al., 2016). Therefore, the limited supply and the rising demand bringing about rising feed prices in aquaculture industry, environmental pressures to use more sustainable lipid resources, and restrictions on contaminants in feeds demand that, for aquaculture sustainable development, alternative lipid sources to fish oil are required urgently (Tocher, 2009). The proposed lipid sources includes vegetable oil sources such as palm oil (PO), rapeseed oil (RO), soybean oil (SO), and linseed oil (LO). Also, marine oil especially krill oil (KO), which extracted from Antarctic krill, containing a high proportion of EPA, DHA and astaxanthin, have the potential to be an effective lipid source in aquaculture feed (Ulven and Holven, 2015). However, these lipid sources mentioned above have different fatty acid profiles, thus respond differently to genes involved in lipid metabolism including anabolism and catabolism. The variation in the fatty acid profiles of different lipid sources and diets may result in different reactions through the modulation of gene expression of various lipid metabolic enzymes (Price et al., 2000; Ayisi et al., 2018). Many key metabolic enzymes and transcriptional factors play crucial roles in lipogenesis and lipolysis, such as fatty acid synthase (FAS), lipoprotein lipase (LPL), carnitine palmitoyltransferase I (CPTI), sterol regulatory element-binding protein 1 (SREBP1) and many others (Zheng et al., 2013). In addition, long chain fatty acid transporters including fatty acid binding protein (FABP) and scavenger receptor B2 (SR-B2) also play a key role in transporting fatty acid (Nickerson et al., 2009). Most researches related to the effects of lipid sources on lipid metabolism have focused on fish such as *Acanthopagrus schlegelii* (Jin et al., 2017), *Ctenopharyngodon idellus* (Yu et al., 2018), *Larmichthys crocea* (Qiu et al., 2017), *Oncorhynchus mykiss* (Fickler et al., 2018), and *Scophthalmus maximus* L. (Peng et al., 2014). However, a few researches related to lipid metabolism have concentrated on crustaceans, such as *Eriocheir sinensis* (Wei et al., 2017; Liu et al., 2018), *Litopenaeus vannamei* (Chen et al., 2015b), and *Sagmariasus verreauxi* (Shu-Chien et al., 2017). Up to now, researches on this topic still limited.

Lipids which stored in the hepatopancreas are used to produce energy for molting, starvation, limb regeneration in the growth stage of crustaceans through β-oxidation, a major pathway of fatty acid catabolism takes place in mitochondria and peroxisome in the cells (Tocher, 2003). Mitochondria, double-membrane-bound organelle which known as “the powerhouse of the cell,” play the most prominent role in producing the energy currency of the cell, adenosine triphosphate (ATP) (Huang et al., 2010; Parihar et al., 2015). Mitochondrial sirtuins which contain three types (namely Sirt3, Sirt4, and Sirt5) are a highly conserved family of proteins and metabolic sensors of cell’s energetic status that regulate cellular physiology and energy demands in response to metabolic inputs, including fatty acid input (Osborne et al., 2014; Parihar et al., 2015). These sirtuins regulate mitochondrial metabolic functions mainly through controlling post-translational modifications of mitochondrial protein. Sirt3, the predominant mitochondrial deacetylase, is in charge of the deacetylation in many proteins involved in the pathway of mitochondrial energy metabolism, especially the protein complexes of electron transport chain (ETC) (Ahn et al., 2008; Huang et al., 2010; Parihar et al., 2015). Furthermore, Sirt3 can also regulated the mitochondrial biogenesis (Parihar et al., 2015). The levels of Sirt3 are highly responsive to the prevailing nutrient availability of the cell (Osborne et al., 2014). To date, perhaps only very limited studies on the relationships between dietary lipid sources and mitochondrial energy metabolism in crustaceans were conducted.

Swimming crab (*Portunus trituberculatus*), one of the most important commercial mariculture crustacean species, is widely distributed in the coastal waters of China, Japan, Korea, and Malaysia (Jin et al., 2016). Over the past decade, researches on the effects of different dietary lipid sources on growth, tissue fatty acid compositions, immunity and antioxidant capacity and lipid metabolism in crustaceans have increased significantly (Ramesh and Balasubramanian, 2005; Zhou et al., 2007; Unnikrishnan et al., 2010; Hu et al., 2011; Li et al., 2011; Chen et al., 2015a; Han et al., 2015; Shu-Chien et al., 2017). However, researches related to the lipid and energy metabolism on crustaceans are still relatively few. Thus, we carried out a feeding trial to explore the impacts of the different dietary lipid sources (FO, KO, PO, RO, SO, and LO) on the tissue lipid classes, the expression of some vital lipid metabolism and mitochondrial energy metabolism relevant genes for juvenile swimming crab. This study will provide a novel insight into the influences of dietary lipid sources on the lipid metabolism and energy metabolism in crustaceans.

## Materials and Methods

### Diet Preparation and Feeding Trial

**FIGURE 1:**
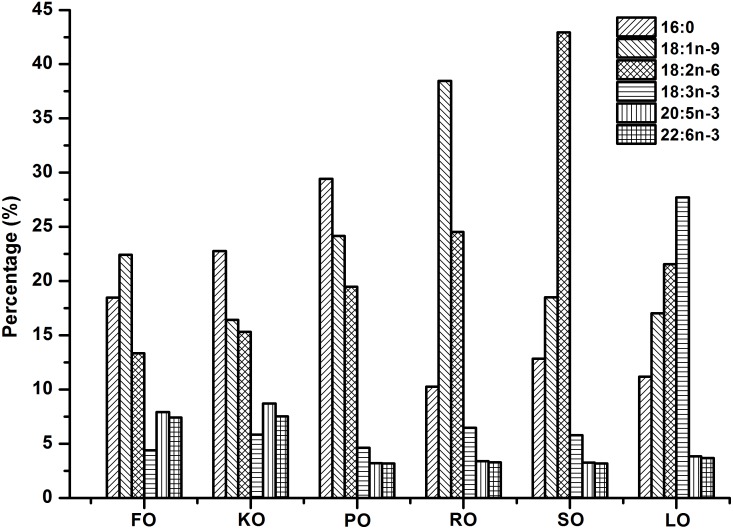
Major fatty acid compositions of different experimental diets (% total fatty acids).

Six isonitrogenous (45% crude protein) and isolipidic (8% crude lipid) experimental diets were formulated to meet the nutrient requirements of swimming crab juveniles based on NRC recommendations (Table 1). The major fatty acid compositions of experimental diets containing different lipid sources (% total fatty acids) are presented in Figure 1. FO diet contained 7.94% EPA and 7.42% DHA of TFA (total fatty acids), KO diet contained 8.71% EPA and 7.54% DHA of TFA, PO diet contained 29.42% C16:0 of TFA, RO diet contained 38.45% C18:1n-9 of TFA, SO diet contained 42.93% C18:2n-6 of TFA, and LO diet contained 27.73% C18:3n-3 of TFA, respectively. All experimental diets were sealed in vacuum-packed bags and stored at -20°C until used to maintain good quality for the feeding trail. Disease-free and similar sized swimming crab juveniles were obtained from Xiangshan crab field (Ningbo, China). The feeding trial was conducted in Ningbo Marine and Fishery Science and Technology Innovation Base (Ningbo, China) located at (121.7784°E, 29.6481°N). Prior to the start of the feeding trial, swimming crab juveniles which caught from the pond were acclimated in the cement pool for 1 week and fed with a commercial diet (45% crude protein, 80% crude lipid, Ningbo Tech-Bank Feed Co., Ltd., Ningbo, China). A total of 270 swimming crab juveniles with an initial weight of 5.43 ± 0.03 g were randomly divided into six diets. Each diet had three replicates, each consisted of 15 crabs. Each crab juvenile was assigned to an individual rectangle plastic basket (35 cm × 30 cm × 35 cm) in the cement pool (6.8 m × 3.8 m × 1.7 m, length × width × depth). The plastic basket had two compartments, one section filled with sand (diameter, <0.5 mm; thickness, 5.0–7.0 cm) to mimic the habitat of swimming crab and the other section as the feeding area. Fifteen plastic baskets were placed in a line next to each other in the cement pool provided with continuous aeration through an air stone to maintain dissolved oxygen levels near saturation levels. All of the crabs were fed experimental diets once daily (daily ration was about 6–8% of wet weight) at 17:00 h. Crab in each plastic basket was weighted once every 2 weeks and the feed’s daily ration was adjusted accordingly. Feces and uneaten feed were removed and about 60% of seawater in the cement pool were daily exchanged to maintain water quality every morning. Seawater temperature in the cement pool was entirely consistent among all individual rectangle plastic basket used for the experiment. Seawater temperature in the cement pool gradually decreases from 32.3 to 26.2°C with the conduct of feeding trial (8 weeks, from July 25th to September 11th). The variation of 6°C referred to the natural temporal variation of temperature over the duration of the experiment. The salinity ranged between 25.5 and 28.6 g/L, pH was 7.3–7.9, ammonia nitrogen was lower than 0.05 mg/L, and dissolved oxygen was not less than 6.0 mg/L during the feeding trail. Salinity, pH, ammonia nitrogen and dissolved oxygen were measured by YSI Proplus (YSI, Yellow Springs, OH, United States). The feeding trial lasted for 8 weeks.

**Table 1:** Formulation and proximate composition of experimental diets (dry matter).

Ingredient (g/100 g)	Dietary lipid sources					
	
	FO	KO	PO	RO	SO	LO
Peru fish meal	15.00	15.00	15.00	15.00	15.00	15.00
Soybean protein concentrate	26.00	26.00	26.00	26.00	26.00	26.00
Soybean meal	20.00	20.00	20.00	20.00	20.00	20.00
Krill meal	3.00	3.00	3.00	3.00	3.00	3.00
Wheat flour	23.50	23.50	23.50	23.50	23.50	23.50
Fish oil^a^	2.00					
Krill oil^b^		2.00				
Palm oil^c^			2.00			
Rapeseed oil^d^				2.00		
Soybean oil^e^					2.00	
Linseed oil^f^						2.00
Soybean lecithin^g^	3.00	3.00	3.00	3.00	3.00	3.00
Vitamin premix^h^	1.00	1.00	1.00	1.00	1.00	1.00
Mineral premix^h^	1.50	1.50	1.50	1.50	1.50	1.50
Ca(H_2_PO_4_)_2_	1.50	1.50	1.50	1.50	1.50	1.50
Choline chloride	0.30	0.30	0.30	0.30	0.30	0.30
Sodium alginate	3.20	3.20	3.20	3.20	3.20	3.20
Proximate composition						
Prude protein	46.42	46.70	46.61	46.52	46.68	46.70
Crude lipid	7.80	7.81	7.79	7.79	7.84	7.80
Moisture	12.59	12.93	12.61	12.04	12.76	12.90
Ash	9.54	9.66	9.54	9.60	9.59	9.54


*^*a*^Fish oil was purchased from Ningbo Tech-Bank Feed Co., Ltd., Ningbo, China. ^*b*^Krill oil was purchased from Kangjing Marine Biotechnology Co., Ltd., Qingdao, China. ^*c*^Palm oil was purchased from Longwei grain oil industry Co., Ltd., Tianjin, China. ^*d*^Rapeseed oil was purchased from Yihai Kerry Co., Ltd., Shanghai, China. ^*e*^Soybean oil was purchased from Yihai Kerry Co., Ltd., Shanghai, China. ^*f*^Linseed oil was purchased from Longshan farm agricultural development Co., Ltd., Gansu, China. ^*g*^Soybean lecithin was purchased from Ningbo Tech-Bank Feed Co., Ltd., Ningbo, China. ^*h*^Vitamin premix and mineral premix were based on Jin (Jin et al., 2016).*

### Sample Collection

In the present study, all procedures complied with Chinese law pertaining to experimental animals. The protocol was approved by the Ethic-Scientific Committee for Experiments on Animals of Ningbo University.

At the end of the experiment, crab in each plastic basket of each replicate was counted and weighted to determine survival, percent weight gain (PWG) and molting ratio (MR). The growth performance are shown in Supplementary Figure S1. Hemolymph samples were taken from the pericardial cavity of six crabs in each replicate and using 1-ml syringes, and collected into 1.5 ml Eppendorf tubes. The hemolymph samples were centrifuged at 3500 rpm for 10 min at 4°C by centrifuge (Eppendorf centrifuge 5810R, Germany). Then, the supernatant was collected, packaged, and stored at -80°C until analysis of serum lipid classes. A large portion of the hepatopancreas was collected and stored at -20°C for the determination of lipid content and lipid classes analysis. A small portion of the hepatopancreas was collected, immediately placed in liquid N_2_, and stored at -80°C for gene expression analysis (eight crabs from each replicate). Another small fresh portion of the hepatopancreas was used to isolate mitochondria and further analysis.

### Lipid Content and Lipids Classes

Lipid content of hepatopancreas was determined gravimetrically after extraction in CHCl_3_/CH_3_OH (2:1) and evaporation to constant weight with a rotating evaporator (IKA RV10, Germany) according to the method described by Folch et al. (1957). Methanol (CH_3_OH, ≥99.7%, CAS 67-56-1) and chloroform (CHCl_3_, ≥99.0%, CAS 67-66-3) were of analytical grade which purchased from Sinopharm Chemical Reagent Co., Ltd. (Shanghai, China). The triacylglycerol (TG) and total cholesterol (TC) contents in serum were assayed using an automatic biochemistry analyzer (VITALAB SELECTRA Junior Pros, Netherlands). The reagent kits for automatic biochemistry analyzer were purchase from Biosino Bio-technology and Science Inc. (Beijing, China). TG and TC levels in hepatopancreas were determined using Triglyceride GPO-PAP and Cholesterol CHOD-PAP kits (Nanjing Jiancheng Bioengineering Institute, China). High-density lipoprotein cholesterol (HDL-C) and low-density lipoprotein cholesterol (LDL-C) concentrations were determined using a commercial assay kit (Nanjing Jiancheng Bioengineering Institute, China) based on a modification of the cholesterol oxidase method described by Rifai et al. (2000). HDL-C and LDL-C reacted with cholesterol oxidase and cholesterol esterase in the presence of chromogens to produce a colored product (Chen et al., 2007). The concentrations of free fatty acids (FFA) in the serum and hepatopancreas were determined using a commercial assay kit (Nanjing Jiancheng Bioengineering Institute, China) by the method of Duncombe. In brief, FFA reacted with copper reagents to form Cu^2+^ salt, then Cu^2+^ salt reacted with the chromogen diethyldithiocarbamate to give a yellow color (Duncombe, 1963). Each determination was performed in triplicate.

### Isolated Mitochondria Preparation

A small fresh portion of the hepatopancreas was used to isolate mitochondria by the method as previously described (Bustamante et al., 1977). Briefly, the tissue fragments were minced by careful shearing, rinsed to remove residual impurities with normal saline, weighed (≈200 mg), put into an ice-cold isolation buffer containing 0.25 M sucrose, 10 mM Tris–HCl, and 0.5 mM EDTA at pH 7.4, and then gently homogenized at 1000 g for 10 min; the supernatant was then centrifuged at 10,000 g for 10 min. The mitochondrial pellets were collected, washed twice and then resuspended in the isolation buffer. The pellets were stored at -80°C. All of the operations were carried out on ice. The mitochondrial protein concentrations were determined using a BCA protein assay kit according to manufacturer’s protocol (Beyotime Biotechnology, Shanghai, China).

### Mitochondrial Membrane Lipids and Fatty Acid Compositions

Total lipids were extracted from mitochondria (10 mg protein) followed the method of Bligh and Dyer with few modifications (Bligh and Dyer, 1959). The extracts were dried under N_2_ flow and resuspended in 1 ml CHCl_3_/CH_3_OH/HCOOH (1:1:0.1, v/v/v). The mixture was added 0.5 ml 1 M KCl/2 M H_3_PO_4_ and after shaking for 30 s; the mixture was then centrifuged at 4500 *g* for 5 min. Formic acid (HCOOH, ≥98.0%, CAS 64-18-6) and phosphoric acid (H_3_PO_4_, ≥85.0%, CAS 7664-38-2) were of analytical grade which purchased from Sinopharm Chemical Reagent Co., Ltd. (Shanghai, China). The mixture separated into two layers and the subnatant was transfered to a new tube. Added 0.5 ml CHCl_3_/CH_3_OH (2:1, v/v) to the new tube and the mixture was evaporated to constant weight using a rotating evaporator. The evaporated substance was mitochondrial membrane lipid fatty acid, then collected into the tube, added 200 μl CHCl_3_/CH_3_OH (2:1, v/v) to be dissolved and stored at -20°C for fatty acid analysis. The fatty acid compositions were determined using the method described by Zuo et al. (2013) with some modifications. All of the solvents contained 0.005% (w/v) of *tert*-butylhydroxytoluene (BHT) to prevent the oxidation of PUFAs. Diets (approximately 100 mg) and mitochondrial membrane lipid fatty acid solutions (approximately 100 μl) were thawed at 4°C, then added to a 12 ml volumetric glass screwed tube with lid containing a teflon gasket. Followed by adding 3 ml KOH-CH_3_OH (1 N) and heated at 75°C in a water bath for 20 min. After cooling, 3 ml HCL–CH_3_OH (2 N) was added and the mixture was incubated at 75°C in a water bath for another 20 min. Previous tests were conducted to ensure that all fatty acids can be esterified. Finally, 1 ml hexane was added into the mixture above, shaken vigorously for 1 min, added 1 ml ultra-pure water to promote layering, and then collected supernatant into the ampoule bottle. The solvent contained the FAMEs (fatty acid methyl esters) in the ampoule bottle was reduced to dryness by termovap sample concentrator, and the FAMEs were resuspended in 500 μL of *n*-hexane and stored at -20°C until used for gas chromatography (GC) analysis. HPLC-grade *n*-hexane (≥95.0%, CAS 110-54-3) and the standard mixtures of 37 FAMEs were purchased from Sigma (St. Louis, MO, United States). All FAMEs were separated and analyzed on a gas chromatograph mass spectrometer (GC-MS) (Agilent-GCMS 7890-5975C; Agilent Technologies, Santa Clara, CA, United States). The GC column was a capillary HP-5MS column (Agilent Technologies, Santa Clara, CA, United States). The column length was 30 m with an internal diameter of 0.25 mm. The film thickness was 0.25 μm. Mass spectra were scanned from m/z 50–800. Peaks and fatty acids were identified using retention times from standards by comparing the mass spectra with a commercially available standard library (National Institute of Standards and Technology Mass Spectral Library 2011). Results were presented as relative percentages of each fatty acid (% total fatty acids), calculated using the peak area ratio.

### Total RNA Extraction, Reverse Transcription, and Real-Time Quantitative PCR

RNA extraction, cDNA synthesis, and real-time quantitative PCR were performed based on the procedures described by Jin et al. (2017). Briefly, total RNA was extracted from hepatopancreas samples with TRIzol reagent (Takara, Japan) following the manufacturer’s protocol and the RNA was treated with RNase-Free DNase (Takara, Japan) to remove DNA contamination. The cDNA was generated from 1000 ng of DNase treated RNA and synthesized by a Prime Script^TM^ RT Reagent Kit with gDNA Eraser (Takara, Japan) using Mastercycler nexus GSX1 PCR (Eppendorf, Germany). Real-time quantitative PCR was conducted by a quantitative thermal cycler (Lightcycler 96, Roche, Switzerland). The complete mitochondrial DNA sequence for swimming crab (*P. trituberculatus*) has been determined as previously described (Yamauchi et al., 2003). All primers were synthesized by BGI (The Beijing Genomics Institute, Shenzhen, China). Specific primers for the candidate genes *fas* (fatty acid synthase), *acc* (acetyl-CoA carboxylase), *g6pd* (glucose 6-phosphate dehydrogenase), *6pgd* (6-phosphogluconate dehydrogenase), *lpl* (lipoprotein lipase), *hsl* (hormone-sensitive lipase), *cpt1* (carnitine palmitoyltransferase 1), *cpt2* (carnitine palmitoyltransferase 2), *fabp1* (fatty acid binding protein 1), *fatp4* (fatty acid transport protein 4), *srb2* (scavenger receptor class 2), *srebp-1*, (sterol regulatory element-binding protein-1), *hnf4*α, (hepatocyte nuclear factor 4-alpha), *nd*, (NADH dehydrogenase), *sdhc*, (succinate dehydrogenase complex, subunit C), *cytb* (cytochrome b), *cox* (cytochrome c oxidase), *sirt* (silent information regulator), *nrf1* (nuclear respiratory factor 1), and β-actin used for qPCR were designed by Primer Premier 5.0 (Supplementary Table S1). β-actin was used as a house-keeping gene and the stability of β-actin was confirmed (Pan et al., 2010). The fluorescence data acquired were normalized to β-actin based on the 2^-ΔΔt^ method (Livak and Schmittgen, 2001). The relative mRNA expression of target genes in crabs fed FO was selected as the calibrator.

### Statistical Analysis

The results are expressed as the means ± SEM (*n* = 3). Data were processed using one-way analysis of variance (ANOVA), followed by Tukey’s test. Firstly, all the data were tested for normal distribution and homogeneity of variance. Then, the group means could be further compared using Tukey’s multiple range test. All statistical analysis were performed using SPSS 22.0 (SPSS, Chicago, IL, United States). A value of *P* < 0.05 was considered statistically significant. OriginPro 8.5 software (OriginLab, Los Angeles, CA, United States) was used for figure processing.

## Results

### Growth

Supplementary Figure S1 shows the effects of different lipid sources on growth of juvenile swimming crab. There were no statistical differences in survival of swimming crab juveniles among all diets (*P* > 0.05). It was found that crabs fed KO diet had a significantly higher PWG (percent weight gain) and MR (molting ratio) than those fed other diets (*P* < 0.05), followed by FO diet.

### Lipid Content in Hepatopancreas

**FIGURE 2:**
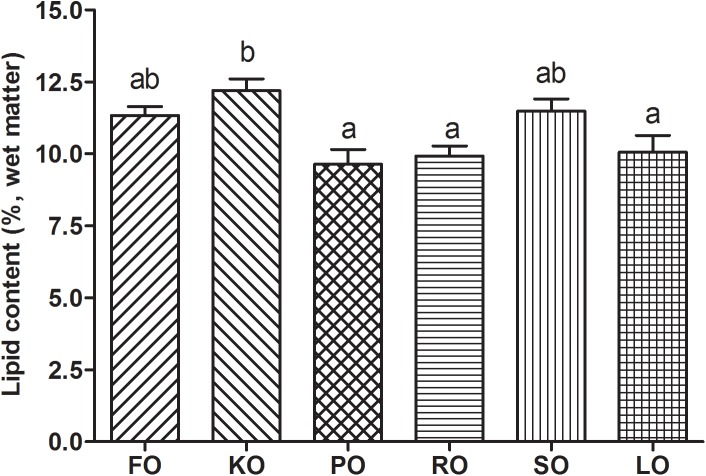
Lipid contents (%, wet matter) in hepatopancreas of swimming crab juveniles fed different lipid sources. Letters on the top of columns sharing a common letter are not significantly different (*P* ≥ 0.05).

Figure 2 shows the lipid contents (%, wet matter) in hepatopancreas of swimming crab juveniles fed different lipid sources. Crabs fed KO had a significantly higher lipid content in hepatopancreas than that fed diet supplemented with PO, RO and LO (*P* < 0.05), while no statistic differences were found compared to FO and SO diets.

### Lipid Classes in Serum and Hepatopancreas

Table 2 compares the lipid classes levels in serum and hepatopancreas of swimming crab fed with different diet supplements. The levels of TG and TC in serum were not affected by different dietary lipid sources (*P* > 0.05). FO diet had a significantly higher HDL-C levels in serum than other diets (*P* < 0.05). The highest level of LDL-C in serum was found in RO diet, while PO diet had the lowest one. The FFA concentrations in serum of crabs fed KO was significantly more abundant than that fed other diets (*P* < 0.05). Different dietary lipid sources could also significantly affect the lipid classes concentrations in hepatopancreas (*P* < 0.05). Hepatopancreas TG concentrations in crabs fed diets supplemented with RO and SO were significantly higher than that fed other diets (*P* < 0.05). The content of TC in FO diet was the lowest, while SO diet had the highest content of TC among all diets. Crabs fed diet supplemented with KO had a high level of LDL-C as well as HDL-C. However, it was found that FO diet had a high level of HDL-C, but a low level of LDL-C. The FFA concentrations in hepatopancreas of crabs fed KO and SO diets were significantly higher than that fed FO and PO (*P* < 0.05).

**Table 2:** Effects of different dietary lipid sources on serum and hepatopancreas lipid classes contents of swimming crab^a^.

Item	Dietary lipid sources					
	
	FO	KO	PO	RO	SO	LO
Serum						
TG (mmol/L)^b^	0.07 ± 0.01	0.09 ± 0.01	0.06 ± 0.02	0.07 ± 0.01	0.06 ± 0.01	0.08 ± 0.01
TC (mmol/L)^c^	0.04 ± 0.01	0.06 ± 0.00	0.05 ± 0.02	0.04 ± 0.02	0.04 ± 0.01	0.05 ± 0.01
HDL-C (μmol/L)^d^	79.15 ± 4.60^b^	49.79 ± 9.64^a^	44.68 ± 1.28^a^	43.02 ± 1.51^a^	45.96 ± 7.97^a^	45.19 ± 1.59^a^
LDL-C (μmol/L)^e^	25.83 ± 2.78^ab^	22.23 ± 2.40^ab^	18.21 ± 1.53^a^	32.18 ± 4.04^b^	25.83 ± 4.88^ab^	21.17 ± 3.05^ab^
FFA (μmol/L)^f^	125.47 ± 16.94^a^	478.62 ± 86.25^c^	116.79 ± 17.84^a^	126.04 ± 5.47^a^	241.69 ± 41.43^ab^	275.02 ± 22.90^b^
Hepatopancreas						
TG (mmol/gprot)	0.10 ± 0.03^a^	0.09 ± 0.02^a^	0.09 ± 0.01^a^	0.16 ± 0.02^b^	0.16 ± 0.02^b^	0.08 ± 0.01^a^
TC (mmol/gprot)	5.64 ± 0.62^a^	20.80 ± 1.56^b^	7.38 ± 1.06^a^	18.82 ± 1.24^b^	31.58 ± 3.68^c^	25.19 ± 1.71^bc^
HDL-C (mmol/gprot)	2.02 ± 0.14^c^	1.20 ± 0.10^abc^	1.12 ± 0.17^ab^	1.41 ± 0.25^abc^	1.68 ± 0.29^bc^	0.57 ± 0.09^a^
LDL-C (mmol/gprot)	0.12 ± 0.01^ab^	0.24 ± 0.02^c^	0.09 ± 0.00^a^	0.15 ± 0.01^b^	0.13 ± 0.01^ab^	0.13 ± 0.01^ab^
FFA (mmol/gprot)	1.49 ± 0.19^a^	2.12 ± 0.22^b^	1.43 ± 0.15^a^	1.81 ± 0.07^ab^	2.08 ± 0.17^b^	1.90 ± 0.23^ab^


*^*a*^Values are means ± SEM of three replicates (*n* = 3). Values within the same row sharing a common letter in superscripts are not significantly different (*P* ≥ 0.05). ^*b*^TG, triglyceride; ^*c*^TC, total cholesterol; ^*d*^HDL-C, high density lipoprotein cholesterol; ^*e*^LDL-C, low density lipoprotein cholesterol; ^*f*^FFA, free fatty acid.*

### Expression of Lipid Metabolism Genes in Hepatopancreas

**FIGURE 3:**
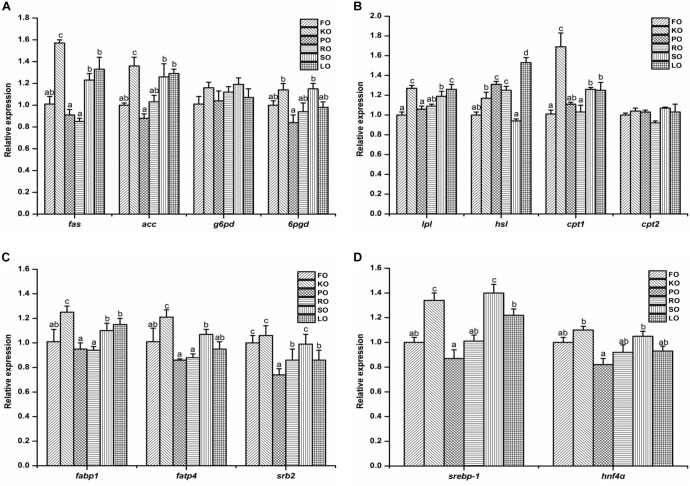
Effects of different dietary lipid sources on relative mRNA expression levels of genes involved in lipid metabolism including anabolism **(A)**, catabolism **(B)**, transport and uptake **(C)**, and transcription factors **(D)** in the hepatopancreas of juvenile swimming crab. The gene expression of the positive control group (FO) was set at 1. Letters on the top of columns with the same fill pattern sharing a common letter are not significantly different (*P* ≥ 0.05). Significant differences at *P* < 0.05 (Tukey’s test). *fas*, fatty acid synthase; *acc*, acetyl-CoA carboxylase; *g6pd*, glucose 6-phosphate dehydrogenase; *6pgd*, 6-phosphogluconate dehydrogenase; *lpl*, lipoprotein lipase; *hsl*, hormone-sensitive lipase; *cpt*, carnitine palmitoyltransferase; *fabp1*: fatty acid binding protein 1; *fatp4*, fatty acid transport protein 4; *srb2*, scavenger receptor class 2; *srebp-1*, sterol regulatory element-binding protein-1; *hnf4*α, hepatocyte nuclear factor 4-alpha.

The relative gene expression of some lipid metabolism pathways in the hepatopancreas of juvenile swimming crab including anabolism (A), catabolism (B), transport and uptake (C), and transcription factors (D) are shown in Figure 3. Among genes related to anabolic pathway (Figure 3A), the expression levels of *fas* and *acc* in crabs fed KO were significantly higher than other diets (*P* < 0.05). The transcript levels of *fas*, *acc*, and *6pgd* in crabs fed PO were all the lowest. There were no significant differences in the expression of *g6pd* in all diets (*P* > 0.05). With regards to the relative expression of genes related to lipid catabolism (Figure 3B), the present results showed that the expression levels of *lpl*, *hsl*, and *cpt1* were significantly affected by different dietary lipid sources (*P* < 0.05), while there were no significant differences in the gene expression of *cpt2* in all diets (*P* > 0.05). Crabs fed KO and LO had significantly higher expression levels of *lpl* than other diets (*P* < 0.05). The highest expression levels of *hsl* and *cpt1* appeared in LO and KO diets, respectively, which were both significantly higher than other diets (*P* < 0.05). Figure 3C shows the relative expression of genes involved in fatty acid transport and uptake. Crabs fed KO had significantly higher gene expression levels of *fabp1* and *fatp4* than that fed other diets (*P* < 0.05). The expression level of *srb2* was up-regulated significantly by dietary FO, KO and SO (*P* < 0.05). Eventually, the expression levels of genes related to transcription factors are presented in Figure 3D. The relative expression levels of *srebp-1* in KO and SO diets were significantly higher than other diets (*P* < 0.05). Crabs fed KO had the highest expression levels of *hnf4*α, followed by SO diet.

### Fatty Acid Compositions of Mitochondrial Membrane Lipid

**FIGURE 4:**
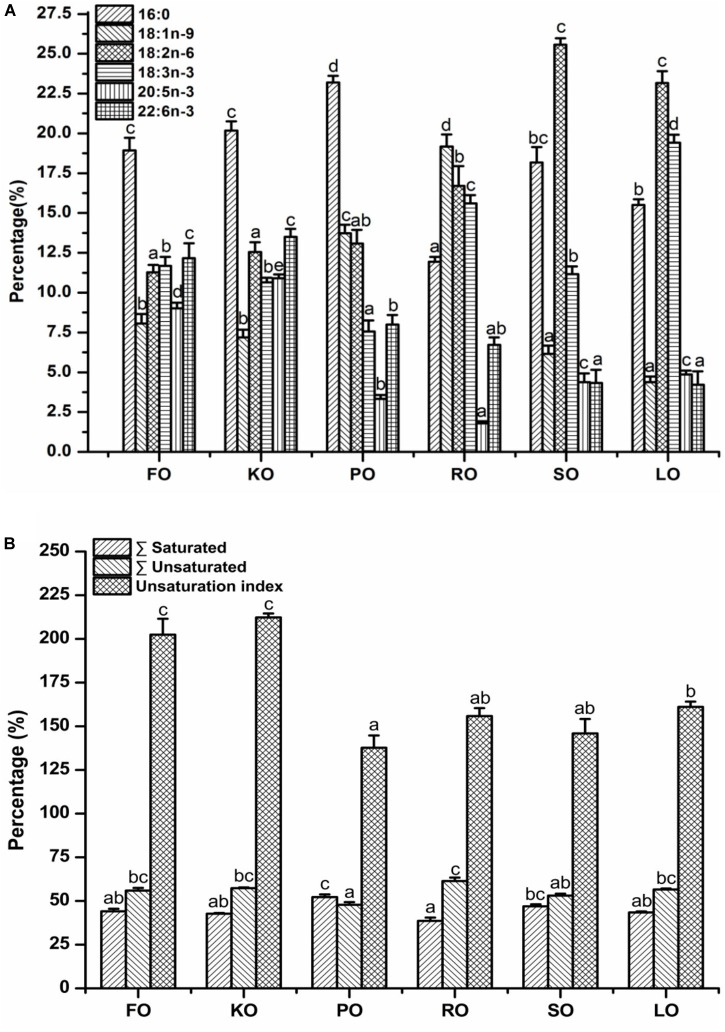
Major fatty acid compositions **(A)** (% total fatty acids) and unsaturation index **(B)** of mitochondrial membrane lipid in hepatopancreas of juvenile swimming crab. Letters on the top of columns with the same fill pattern sharing a common letter are not significantly different (*P* ≥ 0.05).

The major fatty acid compositions (Figure 4A) (% total fatty acids) and unsaturation index (Figure 4B) of mitochondrial membrane lipid in hepatopancreas are presented in Figure 4. In general terms, mitochondrial membrane lipid fatty acid profiles reflected the diets, but there were some differences. Each diet had a particularly characteristic fatty acid in mitochondrial membrane. For instance, the percentage of EPA and DHA in crabs fed FO and KO diets were significantly higher than other diets (*P* < 0.05). Moreover, KO diet had a significantly higher content of EPA than FO diet (*P* < 0.05). The maximum value of palmitic acid (PA, 16:0) in mitochondrial membrane was discovered in PO diet. The highest contents of oleic acid (OA, 18:1n-9) and α-linolenic acid (ALA, 18:3n-3) in mitochondrial membrane were found in RO diet and LO diet, respectively. Crabs fed diets containing SO and LO had a significantly higher proportion of linolenic acid (LA, 18:2n-6) than that fed other diets (*P* < 0.05). From Figure 4B, it was found that the unsaturation index of mitochondrial membrane lipid in FO and KO diets were significantly higher than other diets (*P* < 0.05).

### Electron Transport Chain Complex and Mitochondrial Energy Metabolism

**FIGURE 5:**
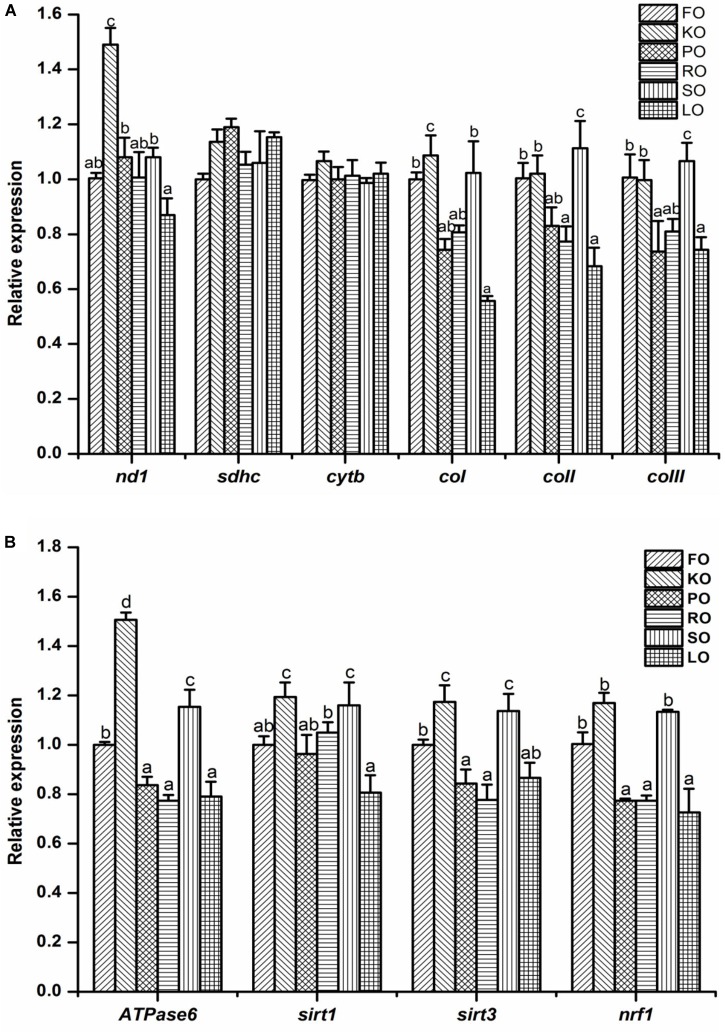
Effects of different dietary lipid sources on relative mRNA expression levels of genes related to electron transport chain complex **(A)** and mitochondrial energy metabolism **(B)** in the hepatopancreas of juvenile swimming crab. The gene expression level of the positive control group (FO) was set at 1. Letters on the top of columns with the same fill pattern sharing a common letter are not significantly different (*P* ≥ 0.05). Significant differences at *P* < 0.05 (Tukey’s test). *nd*, NADH dehydrogenase; *sdhc*, succinate dehydrogenase complex, subunit C; *cytb*, cytochrome b; *cox*, cytochrome c oxidase; *sirt*, silent information regulator; *nrf1*, nuclear respiratory factor.

The electron transport chain complex (Figure 5A) and mitochondrial energy metabolism (Figure 5B) in the hepatopancreas of juvenile swimming crab are shown in Figure 5. The gene expression levels of *nd1* and *coI* in KO diet were significantly higher than other diets (*P* < 0.05). The highest expression level of *coII* appeared in SO diet, and it was significantly higher than other diets (*P* < 0.05), followed by FO and KO diets. Besides, the similar trend was found in expression of *coIII* among all diets. Interestingly, the expression trends of genes related to mitochondrial energy metabolism were similar. In brief, crabs fed diet supplemented with KO had significantly higher expression levels of *Atpase6* than that fed other diets (*P* < 0.05), followed by SO diet, then FO diet. The gene expression levels of *sirt1* and *sirt3* were all significantly up-regulated when crabs fed KO and SO diets, respectively (*P* < 0.05). Crabs fed FO, KO and SO diets had significantly higher expression level of *nrf1* than that fed other diets (*P* < 0.05).

## Discussion

It was reported that different dietary lipid sources affected the expression levels of genes involved in various fatty acid metabolic pathways (Minghetti et al., 2011; Martinez-Rubio et al., 2013). In crustaceans, dietary lipids are mainly assimilated in the hepatopancreas, then being distributed to other tissues through the hemolymph (Coutteau et al., 1997). In the present study, significantly higher hepatopancreas lipid deposition in KO group could be partly due to phospholipid (PL) in krill oil. KO contained a high proportion of EPA and DHA, and 30–65% of the fatty acids were esterified in phospholipids (PLs) (mainly phosphatidylcholine) (Ulven and Holven, 2015). However, fatty acids in vegetable oils and fish oil were mostly esterified in triacylglycerol (TAG) (Ulven and Holven, 2015). Some researches also reported that lipid deposition increased due to PL supplementation in crustaceans, such as *Penaeus japonicus* (Kontara et al., 1997), *Penaeus penicillatus* (Chen and Jenn, 1991), and *P. trituberculatus* (Li et al., 2016). Another reason was that the high expression levels of genes involved in anabolism, such as *fas*, *acc*, *6pgd*, *g6pd*, and *srebp-1* (Ayisi et al., 2018). Some studies demonstrated that FAS is the main lipogenic enzyme which catalyzes successive condensation reactions to form a fatty acid, playing a key role in energy homeostasis (Chen et al., 2015). ACC is a biotin-dependent enzyme that catalyzes the irreversible carboxylation of acetyl-CoA to malonyl-CoA, the rate-limiting step in fatty acid biosynthetic pathway (Barber et al., 2005). 6PGD and G6PD are major regulatory enzymes involved in the production NADPH and play a role in the biosynthesis of fatty acid (Chen et al., 2013). SREBP is a main regulator of fatty acid/lipid and cholesterol biosynthesis (Menoyo et al., 2004). The results of anabolic gene expression showed that crabs fed KO diet up-regulated the gene expression of *fas*, *acc*, *6pgd*, and *srebp-1*, which may suggest that KO could promote the anabolism of lipid and increase the lipid deposition in hepatopancreas of crabs. This is probably the major reason that crabs fed KO diet had higher lipid content in hepatopancreas. Indeed, some studies about fish demonstrated that dietary lipid sources could regulate expression levels of genes involved in the anabolic process, such as *g6pd* and *fas* (Menoyo et al., 2004; Panserat et al., 2008; Morais et al., 2011; Peng et al., 2014). In one research about spiny lobster, the researchers found that the gene expression of *fas* was significantly up-regulated by dietary KO and VO compared to dietary FO, which was similar to present study (Shu-Chien et al., 2017). It was noteworthy that the vegetable oil of previous study about spiny lobster came from linseed and palm oils, which was a blend. The fatty acid composition of VO diet in previous study combines the characteristic of SO and LO diets with the highest levels of LA and ALA. Therefore, similarity in fatty acid composition led to a high expression level of *fas* in VO diet (spiny lobster), KO, SO, and LO diets (swimming crab). In addition to anabolism relevant genes, the expression of genes related to the catabolic process could also be affected by dietary lipid sources, such as *lpl* and *cpt1* (Peng et al., 2014). LPL is considered as an important factor in lipolysis because it might determine how dietary lipids are partitioned for storage or utilization, and it’s a rate limiting enzyme in the provision of fatty acids (Peng et al., 2014; Qiu et al., 2017). The gene expression of *lpl* is regulated differentially according to the nutritional state, hormonal levels and the needs of the tissues for fatty acids (Fielding and Frayn, 1998). The LO and KO diets had a significantly higher expression level of *lpl* than others, followed by SO diet. While, the KO diet had the highest expression level of *cpt1* and was significantly up-regulated. Moreover, the FFA concentration in serum of crabs fed KO also showed the highest, followed by SO and LO diets, which showed a positive correlation between the FFA concentration and the catabolism.

β-oxidation, a major catabolic process of fatty acids, takes place in the cytosol of prokaryotes and the mitochondria of eukaryotes, thus provides acetyl-coenzyme A (acetyl-CoA) as a substrate for the citric acid cycle as well as NADH and FADH_2_ as co-enzymes used in the electron transport chain (ETC) (Houten and Wanders, 2010). Owing to the negative charge carried by FFA, it cannot penetrate any biological membrane (Voet et al., 2008). Whereas, fatty acids bound to albumin can be transferred across the plasma membrane by the action of plasma membrane fatty acid binding proteins (FABP_pm_), fatty acid transport proteins (FATP), fatty acid translocase (FAT/CD36, namely SR-B2), and caveolins (Anderson and Stahl, 2013). The gene expression of FABP was regulated by PUFA. What’s more, FABP can transport and store FA to the mitochondria (Olivares-Rubio and Vega-Lopez, 2016). FATP, a membrane protein, is expressed in tissue which is active in fatty acid metabolism and can effectively promote the transport of long-chain fatty acids (LCFA) (Jeppesen et al., 2012). SR-B2, located on mitochondrial membranes, was found to be involved in the regulation of the rate of cellular fatty acid uptake (Smith et al., 2011). From the result of present study, it revealed that crabs fed diets supplemented with KO and SO had higher gene expression levels of *fabp1* and *fatp4*. The expression levels of *srb2* in FO, KO and SO diets could be up-regulated. The FFA which transported into the mitochondria were used to regulate the key genes and proteins of the energy production.

Mitochondria can produce adenosine triphosphate (ATP) from products of the fatty acid β-oxidation. At the mitochondrial inner membrane, electrons from NADH and FADH2 are transferred from electron donors to electron acceptors, passing through ETC to oxygen eventually (Jonckheere et al., 2012). The above process can release energy, namely oxidative phosphorylation, which is used to form ATP by a series of protein complexes within the inner membrane of the cell’s mitochondria in eukaryotes (Voet et al., 2008). This response was largely driven by the expression of genes encoding subunits of Complex I (NADH dehydrogenase). As we can see from the results, KO and SO diets significantly up-regulated the expression level of Complex I. Some previous studies also provided evidences that krill oil supplementation may stimulate mitochondrial respiratory activity (Mootha et al., 2003; Burri et al., 2011). Previous study had found that a coordinated upregulation of nuclear-encoded genes regulating mitochondrial electron transport, and metabolic rate changes were in associated with the content of PUFA in diet (Hulbert et al., 2005). In addition to an increase of gene expression for subunits of complex I, a change of upregulation in the gene expression of *sirt3* was observed in KO and SO diets in present study. Sirt3 deacetylates and activates various target substrates used in oxidation of fatty acids. Sirt3 is a regulator of mitochondrial energy metabolism, particularly Complex I (Ahn et al., 2008). It is important for maintaining basal ATP levels (Ahn et al., 2008). Aside from Sirt3 modulation and changes in gene expression, mitochondrial membrane fatty acid compositions and membrane fluidity have been proposed to play a role in β-oxidation and energy metabolism (Morash et al., 2008; Longo et al., 2016). The unsaturated degree of membrane lipids affects the fluidity of membrane structure and transmembrane transports (Longo et al., 2016). The result showed that KO diet had the highest unsaturated degree of mitochondrial membrane, which partly explain the higher mRNA expression levels of transmembrane transports proteins. Furthermore, nuclear respiratory factor 1, also known as *nrf1*, encodes a protein that homodimerizes, directly regulates the expression of nuclear genes encoding subunits of the respiratory complexes and indirectly regulating the three mitochondrial-encoded COX subunit genes for respiration, mitochondrial DNA transcription and replication (Gleyzer et al., 2005; Wang et al., 2006). High expression of *nrf1* in KO and SO diets demonstrated a marked effect of KO and SO on the upregulation of genes and pathways involved in hepatopancreas energy metabolism. What’s more, the energy produced by mitochondrial could used to maintain a steady state after molting of crabs, which can partly explain the reason why KO diet has the highest molting ratio among all diets when all of these results were considered together.

## Conclusion

In conclusion, swimming crab fed diet supplemented with KO improved lipid metabolism, increased mitochondrial respiration and strengthen energy metabolism of hepatopancreas. Surprisingly, some of these effects were not observed in swimming crab receiving a diet supplemented with FO, emphasizing the influence that the structural form of n-3 PUFAs (esterified to either PL or TAG) has on exerting a biological response. More researches will be needed to find the direct relationships between physiological metabolism and characteristic fatty acid in swimming crab, which can strengthen the exploitation of lipid sources in aquatic feed and deepen the understanding of the physiological metabolism for the fatty acids utilization in swimming crab. These findings could contribute to optimize feeds for swimming crab during its different growth and developmental stages.

## Ethics Statement

In the present study, all procedures complied with Chinese law pertaining to experimental animals. The protocol was approved by the Ethic-Scientific Committee for Experiments on Animals of Ningbo University.

## Author Contributions

QZ and YY conceived and designed the research. YY and XW conducted the research. YY, PS, and MJ performed the statistical analysis. YY wrote the first draft of the manuscript. All authors contributed to manuscript revision and approved the submitted version.

## Conflict of Interest Statement

The authors declare that the research was conducted in the absence of any commercial or financial relationships that could be construed as a potential conflict of interest.
