# Supplementary material for: Regulation of Dietary Lipid Sources on Tissue Lipid Classes and Mitochondrial Energy Metabolism of Juvenile Swimming Crab, Portunus trituberculatus
Source: Front Physiol. 2019 Apr 24;10:454. doi: 10.3389/fphys.2019.00454 (PMC6491875; doi:10.3389/fphys.2019.00454)
Supplement: Supplementary file 1 [file Data_Sheet_1.doc]

***Supporting information***

Table S1. Real-time quantitative PCR (qPCR) primers for lipid and energy metabolism related genes and β-actin^a^

| gene | forward primer (5'-3') | reverse primer (5'-3') | size (bp) | GenBank reference or publication |
| --- | --- | --- | --- | --- |
| *fas* | CTTCAATACCCACCAAACC | CCTCAATGATGCCAGACAC | 229 | MF537400 |
| *acc* | TCTCAGGGCAACCTTACGCT | CGGGAGGCAGTAACCATTCA | 293 | MF537401 |
| *g6pd* | TGAAAAGGTGAAGGTGCTGA | CGGTGGAGTCATCAAGGTAAC | 125 | MF537402 |
| *6pgd* | GGGTGGAACCTCAACTATGG | CGATAGCCATCATAGAAAGCC | 254 | MF537403 |
| *lpl* | ACAAGACAGACGCAGAGTT | CCTCTCCTAACAAACACCC | 147 | MF537406 |
| *hsl* | GTTCCCATTCTCTCCATTG | ACCACCAGCACTGTCACCT | 159 | MF537408 |
| *cpt1* | GCTTGCCTACTACCGACAC | CCTTGGACATCTTACTGCTC | 155 | MF537407 |
| *cpt2* | TGGGACAAGGTTTTGATAGGC | TGGAGATGATGATGTGGTTGA | 123 | PRJNA432636 |
| *fabp1* | CACTCGCCAGTAGTCAATAGG | TCACTTAGAGAGCAAAGGTCAC | 219 | KU950355 |
| *fatp4* | AAGAATGACCCAATGCGTG | GCCAGCGAAAGGTGTCTC | 178 | PRJNA432636 |
| *srb2* | GGAGTGAAGGACCCTCTGCT | CAAACTCAGCCACTGCGGT | 109 | PRJNA432636 |
| *srebp-1* | GTGATGTGTGCCTTGCGAGT | CCAGGGTTCACCAGTGTAGT | 284 | MF537405 |
| *hnf4α* | CCTGTATCAAAGCCATCGT | CGCTGGAAGGGTTAGAAGA | 170 | PRJNA432636 |
| *nd1* | TATGAAATGGTCTGGGC | CCTTATGATGTGGGTCT | 158 | AB093006 |
| *sdhc* | CGGCTCCTACCCACACTACT | CCCAAATCCCACACCAAG | 179 | KY406169 |
| *cytb* | GGCGTCTTCTCATCTCTGGA | GGGACACACCCACCAAGTTA | 233 | KY406169 |
| *cox1* | TATTGTAAGTCAAGAGTCCG | CTCACAGCATAGAAGGTC | 254 | AB093006 |
| *cox2* | GTGAATAACCCGTCTGT | TAATAATCGGAACCCTG | 141 | AB093006 |
| *cox3* | CAAAGGTTTACGGTGAG | ATAATAGCGTGATGGGC | 228 | AB093006 |
| *Atpase6* | TAGCACTCTCTCTACCTTT | AGCAAGTGTTCCTGGTC | 167 | AB093006 |
| *sirt1* | TTGAGGATGTTGTGAGACTG | GGCGGAAATAATGGATG | 183 | PRJNA432636 |
| *sirt3* | TGGTGATGGTTGGGGCA | TGGGCACAGGTCTTGAG | 182 | PRJNA432636 |
| *nrf1* | CATTGACGGCATCCCCA | CACGACACCTTATGTTTCTGG | 207 | AB093006 |
| *β-actin* | GAAGTAGCCGCCCTGGTTGTG | GGGTCAGAATACCTCGCTTGCTC |  | Pan (Pan et al., 2010) |

^a^*fas*, fatty acid synthase; *acc*, acetyl-CoA carboxylase; *g6pd*, glucose 6-phosphate dehydrogenase; *6pgd*, 6-phosphogluconate dehydrogenase; *lpl*, lipoprotein lipase; *hsl*, hormone-sensitive lipase; *cpt*, carnitine palmitoyltransferase; *fabp1*: fatty acid binding protein 1; *fatp4*, fatty acid transport protein 4; *srb2*, scavenger receptor class 2; *srebp-1*, sterol regulatory element-binding protein-1; *hnf4α*, hepatocyte nuclear factor 4-alpha; *nd*, NAD(H) dehydrogenase; *sdhc*, succinate dehydrogenase complex, subunit C; *cytb*, cytochrome b; *cox*, cytochrome c oxidase; *sirt*, silent information regulator; *nrf1*, nuclear respiratory factor 1.

**Figure S1.** Growth of juvenile swimming crab (*Portunus trituberculatus*) fed with different lipid sources. Data are presented as the mean ± SEM of three replicates (n = 3). Letters on the top of columns sharing a common letter with the same color are not significantly different (*P* ≥0.05). (A) initial body weight (IBW); (B) Percent weight gain (PWG, %) = 100 × [final body weight (g) − initial body weight (g)] / initial body weight (g); (C) Survival (%) = 100 × (final number of crab) / (initial number of crab); (D) Molting ratio (MR) = 2 × the number of molting / (final number of crab + initial number of crab).
